# Supplementary material for: Temperature sensitivity of bat antibodies links metabolic state of bats with antigen-recognition diversity
Source: Nat Commun. 2024 Jul 13;15:5878. doi: 10.1038/s41467-024-50316-x (PMC11245544; doi:10.1038/s41467-024-50316-x)
Supplement: Supplementary file 3 — Reporting Summary [file 41467_2024_50316_MOESM3_ESM.pdf]

nature portfolio

Corresponding author(s): Jordan Dimitrov

Last updated by author(s): Jun 21, 2024

Reporting Summary

Nature Portfolio wishes to improve the reproducibility of the work that we publish. This form provides structure for consistency and transparency in reporting. For further information on Nature Portfolio policies, see our Editorial Policies and the Editorial Policy Checklist.

Statistics

For all statistical analyses, confirm that the following items are present in the figure legend, table legend, main text, or Methods section.

n/a	Confirmed	
☐	☒	The exact sample size (*n*) for each experimental group/condition, given as a discrete number and unit of measurement
☒	☐	A statement on whether measurements were taken from distinct samples or whether the same sample was measured repeatedly
☐	☒	The statistical test(s) used AND whether they are one- or two-sided<br>*Only common tests should be described solely by name; describe more complex techniques in the Methods section.*
☒	☐	A description of all covariates tested
☐	☒	A description of any assumptions or corrections, such as tests of normality and adjustment for multiple comparisons
☐	☒	A full description of the statistical parameters including central tendency (e.g. means) or other basic estimates (e.g. regression coefficient) AND variation (e.g. standard deviation) or associated estimates of uncertainty (e.g. confidence intervals)
☐	☒	For null hypothesis testing, the test statistic (e.g. *F*, *t*, *r*) with confidence intervals, effect sizes, degrees of freedom and *P* value noted<br>*Give P values as exact values whenever suitable.*
☒	☐	For Bayesian analysis, information on the choice of priors and Markov chain Monte Carlo settings
☒	☐	For hierarchical and complex designs, identification of the appropriate level for tests and full reporting of outcomes
☒	☐	Estimates of effect sizes (e.g. Cohen's *d*, Pearson's *r*), indicating how they were calculated

Our web collection on statistics for biologists contains articles on many of the points above.

Software and code

Policy information about availability of computer code

Data collection	TECAN iControl 3.7.3; Biacore control software 3.2.1; Carl Zeiss Microscopy ZEN 2.0; GenePix 4000B Molecular Devices; Applied Biosystems™ Sequence Detection System v. 2.4; FACS Diva 8.0.2.
Data analysis	BIAevaluation 4.1.1; GraphPad Prism v. 10.2.3; GraphPad Prism v. 10.0.2; GraphPad Prism v. 9.4.1; Spotxel software v. 1.7.7; Carl Zeiss Microscopy ZEN 2.0; Microsoft Excel for Mac v. 16.76 and v. 16.80; Origin 6.1; FlowJo 10.8.1; Notepad++ v.8.6.7.

For manuscripts utilizing custom algorithms or software that are central to the research but not yet described in published literature, software must be made available to editors and reviewers. We strongly encourage code deposition in a community repository (e.g. GitHub). See the Nature Portfolio guidelines for submitting code & software for further information.

Data

Policy information about availability of data

All manuscripts must include a data availability statement. This statement should provide the following information, where applicable:

- Accession codes, unique identifiers, or web links for publicly available datasets
- A description of any restrictions on data availability
- For clinical datasets or third party data, please ensure that the statement adheres to our policy

The data that support the findings of this study are available from the corresponding

author upon reasonable request. The raw data are presented as Source data file. References to this file are provided in figure legends and the end of the manuscript.

Research involving human participants, their data, or biological material

Policy information about studies with human participants or human data. See also policy information about sex, gender (identity/presentation), and sexual orientation and race, ethnicity and racism.

Reporting on sex and gender	n/a
Reporting on race, ethnicity, or other socially relevant groupings	n/a
Population characteristics	n/a
Recruitment	n/a
Ethics oversight	n/a

Note that full information on the approval of the study protocol must also be provided in the manuscript.

Field-specific reporting

Please select the one below that is the best fit for your research. If you are not sure, read the appropriate sections before making your selection.

☒ Life sciences ☐ Behavioural & social sciences ☐ Ecological, evolutionary & environmental sciences

For a reference copy of the document with all sections, see nature.com/documents/nr-reporting-summary-flat.pdf

Life sciences study design

All studies must disclose on these points even when the disclosure is negative.

Sample size	For most of the experiments collected sera samples were pooled. Each bat serum pool consist of sera obtained from 3-10 individuals. Exceptionally some experiments with N. noctula were performed by using a large pool (35 individuals). The pools were performed for practical reason as the collection of only small volumes of blood from bats is feasible. Our goal was that pool contain samples from identical individuals in terms of gender, age, physiological status.<br>Birds sera pools consisted of serum of 2-4 individuals.
Data exclusions	No data exclusion was performed in the study.
Replication	Technical replicates in the most of experiments were performed with minimum of two samples. Essential experiments were independently repeated at least two times. All types of repetitions and their number were clearly indicated in the manuscript. The replication attempts were successful.
Randomization	For addressing the research objectives no specific randomization of samples was necessary. The principal goal of the study was to compare the effect of temperature on antibodies obtained from different species. Our data indicated that observed effects were not dependent on intra-species characteristics such as age, gender, or period after hibernation.
Blinding	The study did not require blinding. The effect was validated by applying alternative experimental approaches and by using two different species.

Reporting for specific materials, systems and methods

We require information from authors about some types of materials, experimental systems and methods used in many studies. Here, indicate whether each material, system or method listed is relevant to your study. If you are not sure if a list item applies to your research, read the appropriate section before selecting a response.

Materials & experimental systems

n/a	Involved in the study	
☐	☒	Antibodies
☐	☒	Eukaryotic cell lines
☒	☐	Palaeontology and archaeology
☐	☒	Animals and other organisms
☒	☐	Clinical data
☒	☐	Dual use research of concern
☒	☐	Plants

Methods

n/a	Involved in the study	
☒	☐	ChIP-seq
☐	☒	Flow cytometry
☒	☐	MRI-based neuroimaging

Antibodies

Antibodies used	The immunoreactivities of bat and human antibodies (IgG) as well as antibodies from other mammals mouse, rabbit, goat and cattle, in the study were detected by using recombinant protein-G biotin (PierceTM, Thermo Fisher Scientific, Ref # 21193) and streptavidin conjugated with HRP (Invitrogen, Thermo Fisher Scientific, Ref # S911) or in some cases streptavidin-conjugated to AP (Southern Biotech Ref#7105-04). Bat antibodies were also detected by mouse anti-bat L chain specific antibody BT1-4F10 (Novus Biologicals, Ref# NBP2-23483). For detection of mouse IgG goat Anti-mouse IgG-HRP (Southern-Biotech, Ref# 1030-05) was used. Birds antibodies were detected by Goat anti-chicken IgY-HRP (Southern Biotech, Ref # 6100-05). Alternatively, bat and human IgG were directly coupled with fluorochromes as described in the manuscript.
Validation	The capacity of recombinant protein-G biotin to recognize bat IgG was validated by titration ELISA. Human IgG was used as control. The capacity of goat anti-chicken IgY-HRP (SouthernBiotech, Ref # 6100-05) to recognize IgY from different species of birds was assessed by ELISA, where the serum antibodies were directly coated on the plates and the antibody titrated. Chicken antibodies were used as a control.

Eukaryotic cell lines

Policy information about cell lines and Sex and Gender in Research

Cell line source(s)	Human skin endothelial cell line HMEC-1 (ATTC, CRL-3243).<br>Bat nasal epithelial cells (MmNep) line
Authentication	The human endothelial cell line is a standard one and it is certified by the commercial provider (ATTC, Manassas, VA) . No additional authentication was performed in the host laboratory.<br>The bat cell line was obtained in collaboration. It was generated by immortalization of nasal epithelial cells of Myotis myotis as described in PLoS One 9, e109795 (2014).
Mycoplasma contamination	The cell lines have been tested on regular basis for mycoplasma infection and no contamination was detected.
Commonly misidentified lines<br>(See ICLAC register)	n/a

Animals and other research organisms

Policy information about studies involving animals; ARRIVE guidelines recommended for reporting animal research, and Sex and Gender in Research

Laboratory animals	n/a
Wild animals	Bat species - Myotis myotis, Myotis capaccinii, Myotis blythii and Nyctalus noctula were caught at cave entrances using a harptrap, then they were kept in individual cotton bags until processing. The blood samples were collected uropatagium vein according to animal welfare requirements. Before release each sampled bat was given water and mealworms to compensate for blood loss or considered minimizing handling time to reduce stress in the animals.
Reporting on sex	The phenomenon described in the study did not depend on sex. Therefore, sex was not considered in the study design.
Field-collected samples	The study did not involve experimentation with leaving organisms collected from wild. No animals were collected, transported to or hosted in the laboratory.
Ethics oversight	The collection of blood samples from wild animals (bats) were under ethical permit by Bulgarian Biodiversity Act (No 830/19.09.2020 and No 927/04.04.2022).

Note that full information on the approval of the study protocol must also be provided in the manuscript.

Flow Cytometry

Plots

Confirm that:

☒ The axis labels state the marker and fluorochrome used (e.g. CD4-FITC).

☒ The axis scales are clearly visible. Include numbers along axes only for bottom left plot of group (a 'group' is an analysis of identical markers).

☒ All plots are contour plots with outliers or pseudocolor plots.

☒ A numerical value for number of cells or percentage (with statistics) is provided.

Methodology

Sample preparation	FACS experiments were performed with alive or dead Human skin endothelial cell line HMEC-1 (ATTC, CRL-3243). The cell dead was induced by exposure to 1 mM hydrogen peroxide.
Instrument	FACS LSRFortessaTM
Software	Data acquisition by FACS Diva 8.0.2.<br>Data analyses by FlowJo 10.8.1.
Cell population abundance	Not applicable for the current study as homogeneous cell line was used in the experiments.
Gating strategy	The gating was based on the known size and granularity of HMEC-1 cell line as well as on the cell viability (determined by labeling with Annexin V and PI). The relevant part of the gating strategy is displayed in the manuscript (Fig. 5a).

☒ Tick this box to confirm that a figure exemplifying the gating strategy is provided in the Supplementary Information.
